# Antibody-Mediated Rejection of the Heart in the Setting of Autoimmune Demyelinating Polyneuropathy: A Case Report and Review of the Literature

**DOI:** 10.1155/2012/639284

**Published:** 2012-10-15

**Authors:** Kathryn J. Lindley, Ashwin K. Ravichandran, Joel Schilling, Susan M. Joseph

**Affiliations:** ^1^Division of Cardiology, Washington University School of Medicine, Campus Box 8086, Saint Louis, MO 63110, USA; ^2^Department of Medicine, Washington University School of Medicine, Campus Box 8086, Saint Louis, MO 63110, USA; ^3^Department of Immunology and Pathology, Washington University School of Medicine, Campus Box 8086, Saint Louis, MO 63110, USA

## Abstract

*Background.* Antibody-mediated rejection (AMR) is caused by the production of donor-specific antibodies (DSA) which lead to allograft injury in part via complement activation. The inflammatory demyelinating polyneuropathies (IDP) are inflammatory disorders of the nervous system, involving both cellular and humoral immune mechanisms directed against myelin. *Case Report.* A 58-year-old man five years after heart transplant presented with progressive dyspnea, imbalance, dysphagia, and weakness. Nerve conduction studies and electromyogram were consistent with IDP. Plasmapheresis and high-dose steroids resulted in improvement in neurologic symptoms. Within two weeks, he was readmitted with anasarca and acute renal failure, requiring intravenous furosemide and inotropic support. Echocardiogram and right heart catheterization revealed reduced cardiac function and elevated filling pressures. DSA was positive against HLA DR53, and endomyocardial biopsy revealed grade 1R chronic inflammation, with strong capillary endothelial immunostaining for C4d. Plasmapheresis and intravenous immunoglobulin (IVIG) were initiated. His anasarca and renal failure subsequently resolved, echocardiogram showed improved function off inotropes, and anti-DR53 MFI was reduced by 57%. *Conclusions.* This is an example of a single immune-mediated process causing concurrent IDP and AMR. The improvement in cardiac function and neurologic symptoms with plasmapheresis, IVIG, and high-dose steroids argues for a unifying antibody-mediated mechanism.

## 1. Introduction

Cardiac allograft antibody-mediated rejection (AMR) may occur months to years after transplantation and is associated with significant morbidity and mortality [1]. It is caused by the production of donor specific antibodies (DSA), which leads to allograft injury in part via complement activation [1, 2]. The diagnosis of AMR in transplant patients is supported by tissue pathology demonstrating a paucity of lymphocytes, endothelial cell swelling, and positive capillary staining for C4d or C3d fragments [3, 4]. 

Immune-mediated disorders of the nervous system have also been described, including multiple sclerosis, paraneoplastic syndromes, and the inflammatory demyelinating polyneuropathies (IDP), such as acute inflammatory demyelinating polyneuropathy (AIDP) and chronic idiopathic demyelinating polyneuropathy (CIDP) [5]. AIDP is mediated by both cellular and humoral immune mechanism, and is associated with significant complement and antibody activation against myelin [6]. It has also been associated with the presence of antibodies against heparan sulfates [7]. Early inflammatory lesions tend to have a predominant lymphocytic infiltrate, followed by intense macrophage activation [6]. The mechanism of injury in CIDP is less well defined, but patients often have high titers of IgM antibodies and respond to plasmapheresis [5]. Here we present a case of a patient who developed concurrent AMR and IDP.

## 2. Case Report

**Figure 1:**
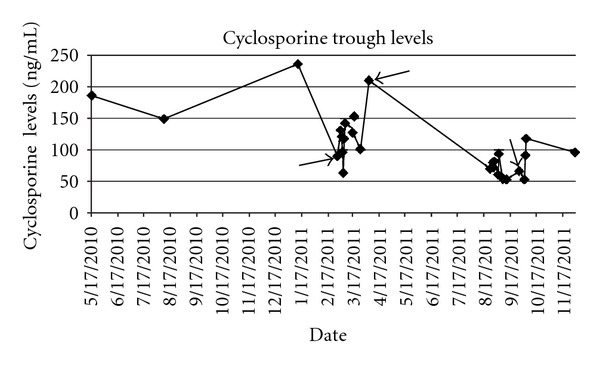
Cyclosporine trough levels.

A 58-year-old African American male with a history of non-ischemic cardiomyopathy who underwent orthotopic heart transplantation 5 years ago presented with increasing shortness of breath, dizziness, and generalized weakness. Six months prior to this presentation he had been diagnosed with cryptococcal meningitis. His initial treatment consisted of amphotericin B and flucytosine, after which he was maintained on chronic suppressive fluconazole therapy. The opportunistic infection also prompted a reduction in his maintenance immunosuppression. Specifically, his mycophenolate mofetil dose was reduced from 750 mg BID to 500 mg BID, and his cyclosporine dose from 125 mg BID to 75 mg BID. His cyclosporine trough was 90 ng/mL on admission, and 142 ng/mL on discharge (Figure 1).

Five months prior to presentation he began to notice bilateral hand numbness and tingling, and, two weeks prior to presentation, he developed imbalance. During his evaluation, the patient was noted to be in atrial flutter with rapid ventricular response, which was initially rate controlled with diltiazem. An echocardiogram performed at that time revealed normal left and right ventricular size and systolic function, with normal diastolic function. 

While in the hospital he developed progressive dysphagia, gait instability, and profound weakness of his bilateral lower extremities. Head CT and brain MRI were unremarkable. Lumbar puncture revealed clear, colorless fluid with an elevated protein level of 147 mg/dL, a normal glucose level of 67 mg/dL, and 5 nucleated cells/mL (within normal limits) of which 85% were lymphocytes. The patient also underwent nerve conduction studies and an electromyogram, which showed conduction block and prolonged distal latencies, suggestive of IDP. Consistent with this diagnosis, he also had a positive IgM antibody to Trisulfated Heparan Disaccharide (TS-HDS). As treatment for IDP, he underwent four sessions of plasmapheresis and was initiated on high-dose oral dexamethasone. The patient had significant improvement in his neurologic symptoms and was discharged one week later to a physical rehabilitation facility.

**Figure 2:**
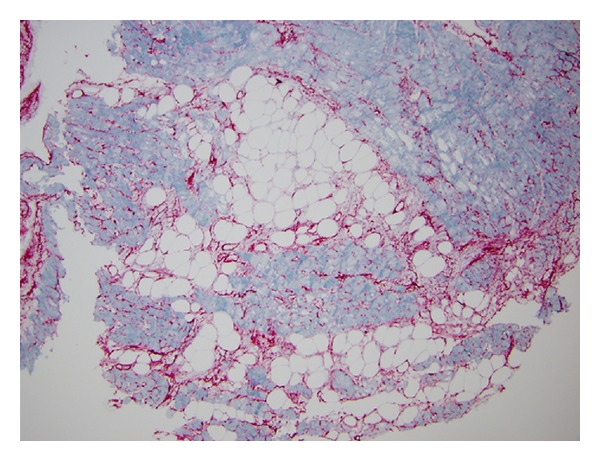
Endomyocardial biopsy.

Within two weeks, the patient was readmitted to the hospital with anasarca. On admission, an echocardiogram demonstrated reduced RV systolic function with an RV tissue doppler S′ of 0.05 m/s_,_ impaired LV diastolic function, and mild global LV systolic dysfunction with an averaged left ventricular outflow tract time-velocity integral (VTI) of 13.75 cm (LVOT VTI was 19.0 cm two weeks earlier). His cyclosporine trough level was 53 ng/mL on admission (Figure 1). Diuresis was attempted with IV furosemide, but the patient rapidly developed acute renal failure. He subsequently underwent a right heart catheterization and endomyocardial biopsy to further evaluate his heart failure (Table 1). Given his elevated right-sided filling pressures, reduced cardiac output, and poor renal function, he was initiated on continuous infusions of dobutamine and furosemide. Endomyocardial biopsy showed ISHLT grade 1R chronic inflammation with strong capillary endothelial immunostaining for C4d, consistent with pAMR2 [4] (Figure 2). DSA against HLA DR53 was detected with a mean fluorescence intensity (MFI) of 5991. 

**Figure 3:**
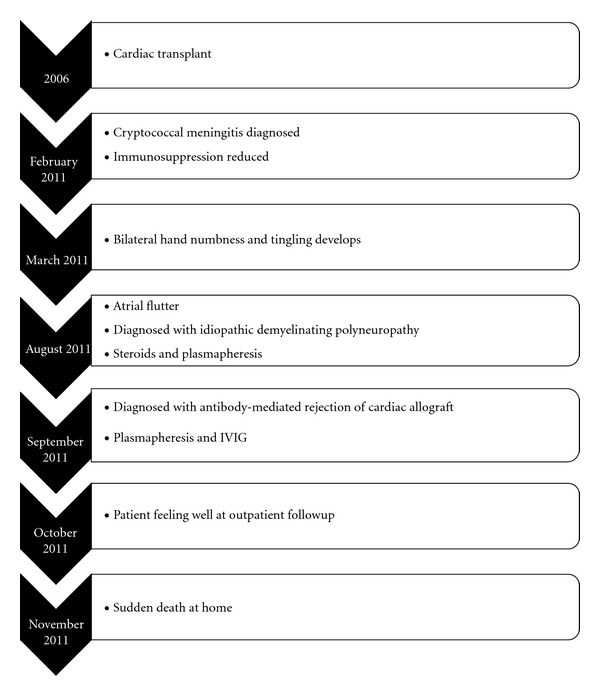
Timeline of events.

The patient was treated with 3 sessions of plasmapheresis followed by intravenous immunoglobulin (IVIG) at a cumulative dose of 1 gram/kilogram. His outpatient immunosuppressive regimen was also continued. After completing plasmapheresis, the patient had a significant improvement in his anasarca and renal function, and his furosemide and dobutamine drips were successfully weaned. A follow-up echocardiogram obtained prior to discharge showed improved systolic and diastolic function. Prior to discharge, the anti-DR53 MFI was reduced to 2585, a 57% reduction compared to initial DSA. At an outpatient follow-up visit one month after discharge, the patient was doing well, without heart failure symptoms and only mild residual weakness. Approximately one month after his follow-up appointment, the patient died suddenly at home (Figure 3). An autopsy was not performed.

## 3. Discussion

This case is an example of concurrent IDP and AMR, a phenomenon not previously described in the literature. While IDP in association with humoral allograft rejection is unique, there has been a report of autoimmune retinopathy associated with chronic renal allograft rejection and a positive antiglomerulus antibody. In that case, the retinopathy resolved concurrently with the resolution of the renal failure via hemodialysis, suggesting that generalized immune activation in response to the renal failure was likely responsible for the retinopathy [8]. In the case of our patient, the anti-HLA DR53 antibody could not be responsible for the neurologic symptoms, since this MHC molecule would only be found on the donor cardiac allograft. Instead, we hypothesize that a more generalized activation of B cells may have contributed to the generation of both the DSA and the anti-TS-HDS antibodies. 

Interestingly, cryptococcal infection has previously been described in association with chronic allograft loss in renal transplant patients [9]. This is thought to occur as a consequence of immune reconstitution syndrome following a reduction or cessation of immunosuppressant agents during the acute infection [9]. It is feasible that the immune response leading to AMR and IDP in our patient was in part related to changes in his immunosuppressive regimen during his recent cryptococcal infection. In support of this notion, his CSA trough was slightly subtherapeutic when he presented with AMR.

In retrospect, the patient's atrial flutter may have been his initial symptoms of AMR, as this arrhythmia has been associated with graft rejection [10]. The duration of plasmapheresis and high-dose steroids were likely sufficient to treat the antibodies responsible for the IDP but may have been insufficient to treat the concurrent AMR, which was as of yet undiagnosed. The significant reduction in anti-HLA DR53 MFI and improvement in clinical and echocardiographic parameters following plasmapheresis and IVIG strongly argue that his heart failure was a consequence of AMR. The resolution of cardiac dysfunction and neurologic symptoms with these therapies suggests the existence of a common underlying antibody-mediated mechanism. Despite an initial response to treatment for AMR, the patient died suddenly at home two months after diagnosis. This highlights the fact that AMR remains a difficult diagnostic and therapeutic dilemma. 

In summary, this is a unique case that demonstrates immune-mediated injury of multiple organ systems, including a transplanted heart allograft. It is possible that both the IDP and AMR resulted from immune activation triggered by recent disseminated cryptococcal infection and/or the subsequent reduction in immunosuppression. This case illustrates that although opportunistic infection is frequently a sign of an overly suppressed immune system, alteration in maintenance immunosuppression should prompt vigilant followup of the patient with a transplanted allograft. 

## Figures and Tables

**Table 1:** Right heart hemodynamics.

Right atrial pressure	16 mmHg
Pulmonary artery pressure	33/22 mmHg
Pulmonary capillary wedge pressure	15 mmHg
Pulmonary oxygen saturation	54%
Cardiac output	4.13 L/min
Cardiac index	1.86 L/min/M^2^
